# Holt-Oram Syndrome With Atrial Septal Defect

**DOI:** 10.7759/cureus.64772

**Published:** 2024-07-17

**Authors:** Shri Lakshmi C.S, Kshitij Sharma, Madhu Manaswini, Keerthi Thakur

**Affiliations:** 1 Pediatrics, Sree Balaji Medical College and Hospital, Chennai, IND

**Keywords:** tbx5, genetic, sporadic, autosomal dominant, left thumb hypoplasia, asd

## Abstract

Holt-Oram syndrome is an autosomal dominant condition marked by heart and upper limb defects. Holt and Oram were the first to narrate this in 1960. Holt-Oram syndrome is the prototype of heart-hand syndromes and has recently been mapped to the long arm of chromosome 12 (12q2). This syndrome was described in 1960 by Dr Mary Holt and Dr Samuel Oram. It is an autosomal dominant condition resulting from a mutation in TBX5 located on chromosome 12q24.1, which regulates cardiac and limb morphogenesis. It must be differentiated from heart-hand syndrome type II (Tobatznik’s syndrome) and heart-hand syndrome type III (MIM No. 140450), which are phenotypically similar. The latter do not map to 12q2, and atrial septal defects do not occur in these conditions. This syndrome is distinguished by heart problems as well as thumb aplasia or hypoplasia. It is sometimes referred to as atriodigital syndrome, upper limb-cardiovascular syndrome, heart-hand syndrome, cardiomelic syndrome, or cardiac limb syndrome. Other upper-limb anomalies include aplasia or hypoplasia of the radius, arm length variation, atypical forearm pronation and supination, uncommon thumb resistance, sloping shoulders, and restricted shoulder movement. All those who are affected have an aberrant carpal bone, which might be the only sign of the illness. Seventy-five percent of those with Holt-Oram syndrome have a congenital cardiac defect, which most frequently affects the septum. In this case, we report a girl who is 4 years and 6 months old and is a known case of Holt-Oram syndrome with an atrial septal defect. She underwent device closure and had come to the pediatric op with fever and cough.

## Introduction

The clinical features of Holt-Oram syndrome, commonly called hand-heart syndrome, are structural defects of the upper limbs and congenital heart issues [1]. Holt-Oram syndrome is a rare disorder with a constellation of preaxial radial ray upper-limb deformities and cardiac septation defects. Diagnosis is based on careful physical examination, imaging, and family history. Molecular genetics, though not available at all places, can be used for confirmation of the disease. Treatment depends on the management of specific symptoms. This article aims to present the classic findings in a female patient with defects in the upper limb and septation of the heart. This report also highlights the diagnostic and management challenges of an uncommon heart-hand syndrome by clinicians in a low-resource setting. Numerous mutations have been reported; however, the *TBX5* gene of the T-box complex, found on chromosome 12, is the one where they occur most frequently. Roughly 40% of instances are due to mutations. This syndrome is usually associated with cardiac structural oddity, most commonly atrial septal defect (ASD), in which secundum is the most common category, heart block of any grade, or may present as either. Lower limb abnormalities rule out the diagnosis, and upper limb abnormalities are invariably present. ASD and ventricular septal defect (VSD) are the two most typical cardiac abnormalities, while others have been documented. Clinical signs can be mild; therefore, the diagnosis might not be made for years or possibly be overlooked.

## Case presentation

A 4-year-6-month-old girl, the third child born to a non-consanguineous marriage, presented with complaints of cough and cold for the past five days and fever for the past two days. She has no other complaints.

She was born full term via a lower segment cesarean section, with a birth weight of 2.75 kg. There was a delayed cry after birth, and she had a history of neonatal intensive care unit (NICU) admission for five days.

Her family history includes a non-consanguineous marriage, with no relevant family history of speech, language, or hearing problem.

Delayed milestones are noted: she achieved head control at five months, sat without support at one year, stood with support at 18 months, and began walking at two years. She mainly expresses her needs through one- to two-word combinations. Her speech is unclear, accompanied by gestures. She occasionally uses three-word combinations and has not yet developed bowel and bladder control.

The child had a history of ASD measuring 12 mm with a left-to-right shunt, for which she underwent device closure in August 2022. She also experienced two episodes of breath-holding spells: the first at one-and-a-half-year old and the second at two years. An MRI revealed dolichocephaly without craniosynostosis and non-restrictive, subcortical predominant T2/flair hyperintensities involving bilateral cerebral hemispheres with no significant white matter volume.

**Figure 1:**
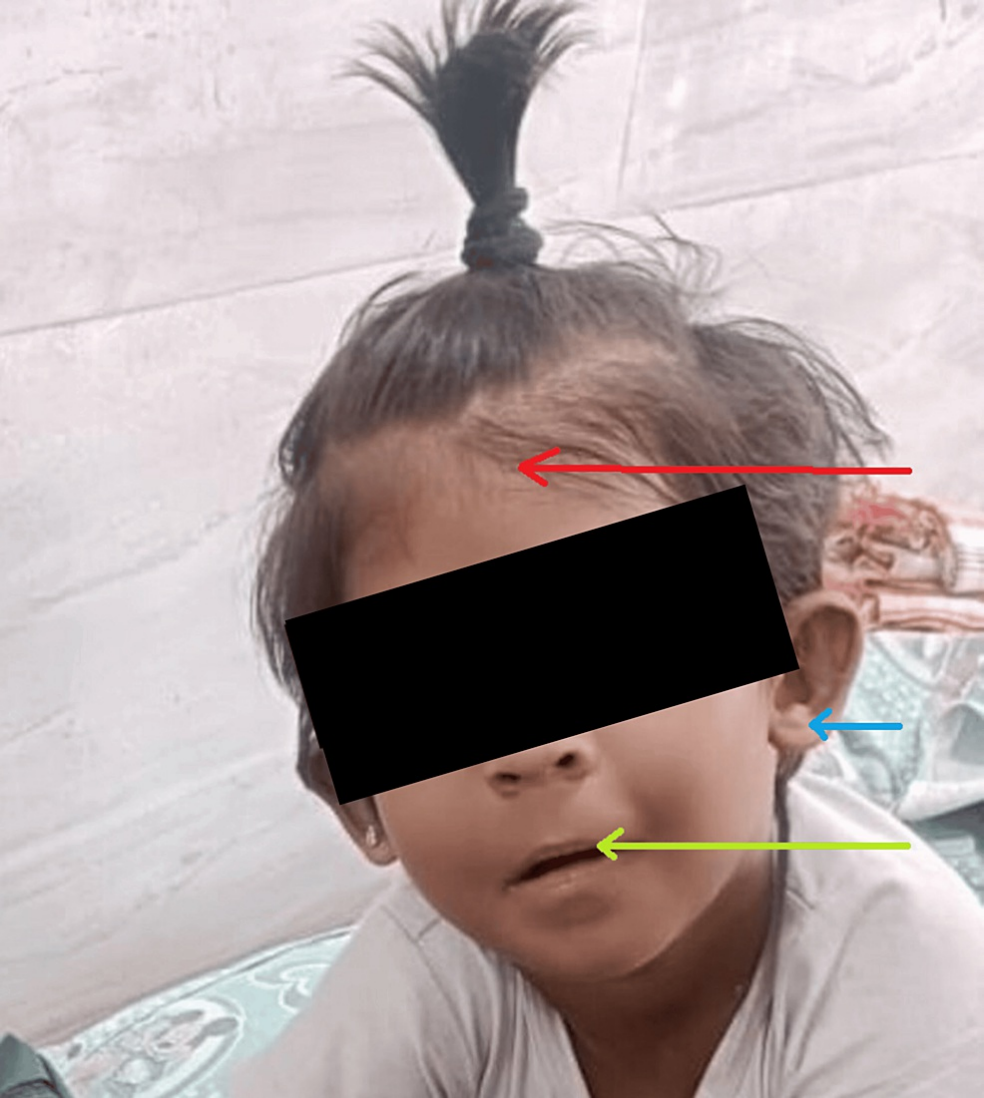
Dysmorphic facies. Red arrow: narrow forehead; blue arrow: low set ears; green arrow: thin lips.

**Figure 2:**
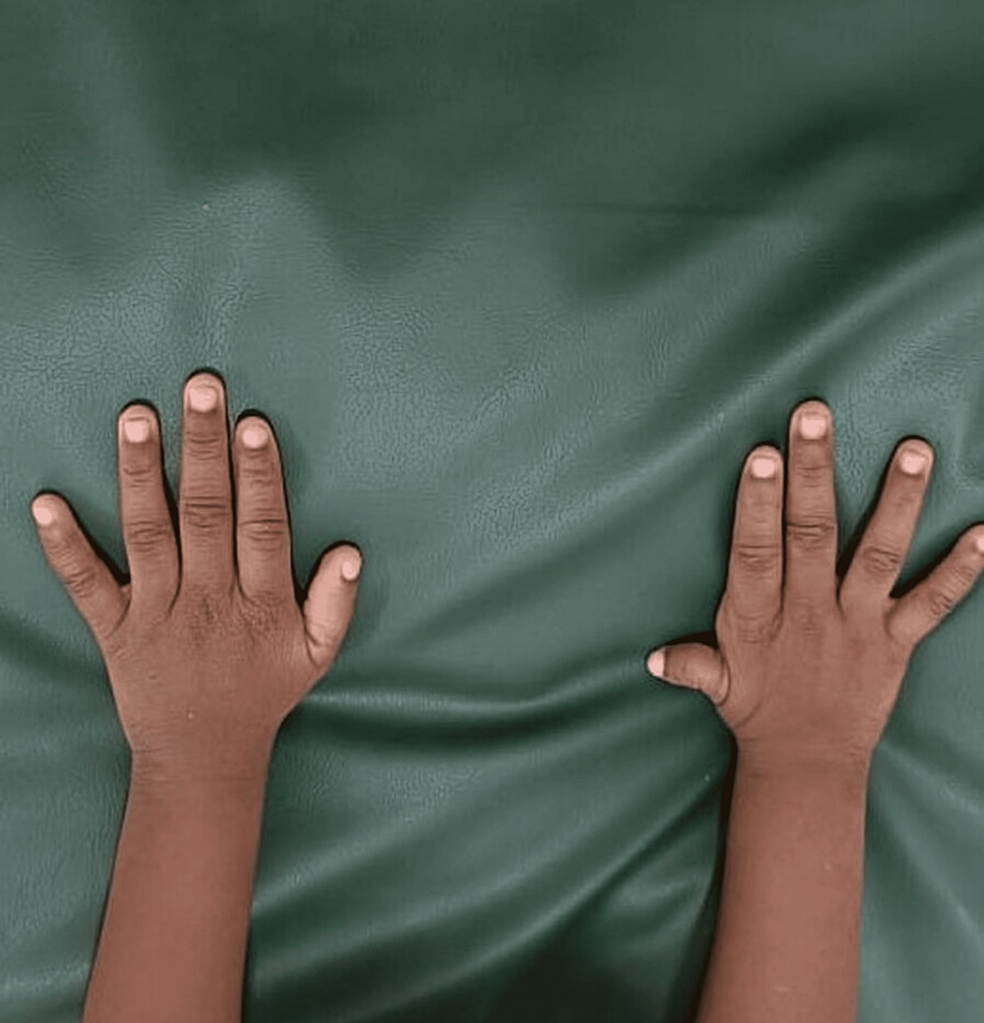
Hypoplastic right thumb.

On examination, the child was active and febrile, with fair hydration and palpable peripheral pulses. There were no signs of pallor, icterus, cyanosis, clubbing, or lymphadenopathy. The child is hyperactive with dysmorphic facies, including narrow forehead, low set ears, depressed nasal bridge, thin lips, retrognathia, and right thumb hypoplasia (Figures 1, 2).

## Discussion

One in 100,000 people is thought to have Holt-Oram syndrome [1]. People who have Holt-Oram syndrome commonly develop upper-limb bone abnormalities. People who are affected have at least one anomaly in their carpal bones, which make up the wrist [2]. Heart issues are present in about 75% of Holt-Oram syndrome patients, and they can be fatal [3]. The most frequent issue is a flaw in the septum, a muscle wall that separates the right and left sides of the heart. ASD refers to a hole in the septum separating the heart's atria (upper chambers), and a VSD is a hole in the septum that divides the heart's ventricles (VSD) [4]. Cardiac conduction illness, which is brought on by flaws in the electrical system that regulates the contractions of the heart chambers, affects certain people with Holt-Oram syndrome [5]. Cardiac conduction disease can cause issues, including a heartbeat that is slower than usual (bradycardia) or a heartbeat that is fast and disorganized (fibrillation). In people with Holt-Oram syndrome, cardiac conduction disease can coexist with other heart defects (such as ASD or VSD) or be the only heart issue. The *TBX5* gene is the only one that has been linked to Holt-Oram syndrome so far [6]. About 74% of people with Holt-Oram syndrome had a *TBX5* gene mutation, according to research.

Holt-Oram syndrome is diagnosed based on physical characteristics and a family history. For abnormalities of the upper limb, hand X-rays are taken. The presence and severity of heart abnormalities and/or cardiac conduction disease are determined using electrocardiography, magnetic resonance imaging, and other imaging modalities. To validate the diagnosis, *TBX5* gene molecular genetic testing is available. A multidisciplinary team of specialists in medical genetics, cardiology, orthopedics, and hand surgery is in charge of management. Arrhythmia treatment may include medication, surgery, and/or pacemaker placement. Individuals suffering from pulmonary hypertension can benefit from pharmacological treatment. Cardiac surgery for congenital heart problems is common. The control of certain symptoms that are noticeable in each person is the goal of Holt-Oram syndrome treatment. The coordinated efforts of a group of professionals may be necessary throughout treatment. Holt-Oram syndrome is an autosomal dominant condition, with approximately 85% of affected individuals having Holy-Oram syndrome as a result of a de novo pathogenic variant. The offspring of an affected individual have a 50% risk of being affected. In pregnancies at 50% risk, a detailed high-resolution prenatal ultrasound examination may detect upper-limb malformations and/or congenital heart malformations.

## Conclusions

It is appropriate to screen seemingly asymptomatic older and younger at-risk relatives of an affected person in order to find those who would benefit from effective cardiac treatment as early as possible. If the TBX5 pathogenic variation in the family is recognized, evaluations may include molecular genetic testing. If the pathogenic variant in the family is unknown, echocardiography, EKG, and hand X-rays may be performed. We present the instance of a patient who was found much too late with an uncommon illness. Even though she displayed signs of both upper limb and heart disorders from birth, a connection between these two conditions was not discovered until much later in life. Pregnant women with Holt-Oram syndrome who have a history of a structural heart defect or heart conduction abnormality should be observed during pregnancy. Affected women who have not had a cardiac evaluation should do so as soon as the pregnancy is detected. In the above presented case, throughout the admission, the patient was under the care of both a cardiologist and a pediatrician. Holt-Oram syndrome is a rare disorder with a constellation of preaxial radial ray upper-limb deformities and cardiac septation defects. Diagnosis is based on careful physical examination, imaging, and family history. Molecular genetics, though not available at all places, can be used for confirmation of the disease. Treatment depends on the management of specific symptoms. This article aimed to present the classic findings in this female patient with defects in the upper limb and septation of the heart. This report also highlights the diagnostic and management challenges of an uncommon heart-hand syndrome by clinicians in a low-resource setting.

## References

[1] Postma AV, van de Meerakker JB, Mathijssen IB (2008). A gain-of-function TBX5 mutation is associated with atypical Holt-Oram syndrome and paroxysmal atrial fibrillation. Circ Res.

[2] Baker EJ, Leung MP, Anderson RH, Fischer DR, Zuberbuhler JR (1988). The cross sectional anatomy of ventricular septal defects: a reappraisal. Br Heart J.

[3] Mori AD, Bruneau BG (2004). TBX5 mutations and congenital heart disease: Holt-Oram syndrome revealed. Curr Opin Cardiol.

[4] Zhu Y, Gramolini AO, Walsh MA (2008). Tbx5-dependent pathway regulating diastolic function in congenital heart disease. Proc Natl Acad Sci U S A.

[5] Reamon-Buettner SM, Borlak J (2004). TBX5 mutations in non-Holt-Oram syndrome (HOS) malformed hearts. Hum Mutat.

[6] Newbury-Ecob RA, Leanage R, Raeburn JA, Young ID (1996). Holt-Oram syndrome: a clinical genetic study. J Med Genet.

